# Exploring How Evidence is Used in Care Through an Organizational Ethnography of Two Teaching Hospitals

**DOI:** 10.2196/10769

**Published:** 2019-03-28

**Authors:** Bryn Lander, Ellen Balka

**Affiliations:** 1 Centre for Clinical Epidemiology Vancouver Coastal Health Research Institute Vancouver, BC Canada; 2 School of Communication Simon Fraser University Burnaby, BC Canada

**Keywords:** clinical practice guidelines, evidence-based medicine, mindlines, ethnography

## Abstract

**Background:**

Numerous published articles show that clinicians do not follow clinical practice guidelines (CPGs). However, a few studies explore what clinicians consider evidence and how they use different forms of evidence in their care decisions. Many of these existing studies occurred before the advent of smartphones and advanced Web-based information retrieval technologies. It is important to understand how these new technologies influence the ways clinicians use evidence in their clinical practice. Mindlines are a concept that explores how clinicians draw on different sources of information (including context, experience, medical training, and evidence) to develop collectively reinforced, internalized tacit guidelines.

**Objective:**

The aim of this paper was to explore how evidence is integrated into mindline development and the everyday use of mindlines and evidence in care.

**Methods:**

We draw on ethnographic data collected by shadowing internal medicine teams at 2 teaching hospitals. Fieldnotes were tagged by evidence category, teaching and care, and role of the person referencing evidence. Counts of these tags were integrated with fieldnote vignettes and memos. The findings were verified with an advisory council and through member checks.

**Results:**

CPGs represent just one of several sources of evidence used when making care decisions. Some forms of evidence were predominately invoked from mindlines, whereas other forms were read to supplement mindlines. The majority of scientific evidence was accessed on the Web, often through smartphones. How evidence was used varied by role. As team members gained experience, they increasingly incorporated evidence into their mindlines. Evidence was often blended together to arrive at shared understandings and approaches to patient care that included ways to filter evidence.

**Conclusions:**

This paper outlines one way through which the ethos of evidence-based medicine has been incorporated into the daily work of care. Here, multiple Web-based forms of evidence were mixed with other information. This is different from the way that is often articulated by health administrators and policy makers whereby clinical practice guideline adherence is equated with practicing evidence-based medicine.

## Introduction

**Figure 1:**
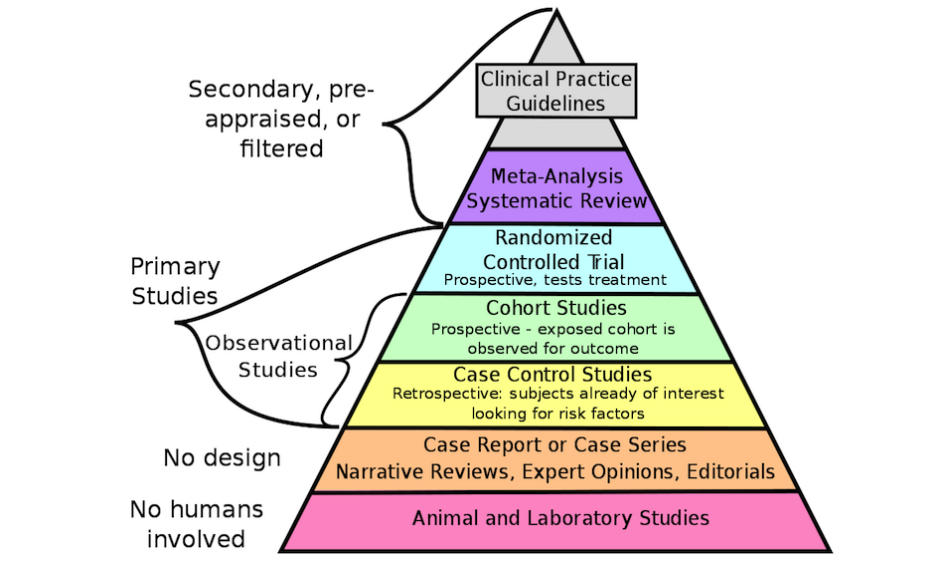
The evidentiary pyramid in evidence-based medicine. Source: “Research design and evidence” by CFCF. Used with permission by Wikimedia Commons (CC BY-SA 4.0, https://creativecommons.org/licenses/by-sa/4.0).

Clinical Practice Guidelines (CPGs) have become ubiquitous, they provide concrete practice recommendations for care providers and are often viewed as an integral component of evidence-based medicine [1-3]. Figure 1 shows an idealized evidence-based medicine hierarchy whereby research is appraised, compared, consolidated, and rewritten into CPGs. Despite the significant resources now devoted to producing, adapting, and implementing CPGs, as highlighted by Eby [4] and others [5,6], CPG adoption in practice has been problematic. Several reviews of existing studies concerned with the underutilization of CPGs focus on barriers to CPG adoption [7-10]. These studies assume that increased CPG uptake leads to more evidence-based care [11]. A few, if any, of these studies considered how information is accessed in the day-to-day work of clinicians.

Gabbay and le May’s ethnographies [12,13] explored how clinicians use research in their day-to-day work. They argue that general practitioners “normally found [it] neither necessary nor helpful to refer to guidelines or other sources of evidence directly during their day-to-day practice” [12]. Instead, clinicians draw on mindlines, which are defined as “collectively reinforced, internalized tacit guidelines” [13]. Mindlines are socially constructed through interactions with health care professionals and patients, personal medical training, experience, and context. They draw on knowledge in a fluid, multidirectional, and context-specific way. This enables clinicians to prioritize relevant information, reduce possible options for action, and decrease clinical uncertainty [11].

Through interviews, Timmermans and Angell [14] found that residents consult an array of evidence including MD Consult, “cheat” books, textbooks, CPGs, review articles, and primary research articles.

Gabbay and le May’s mindlines research was nominated as 1 of 20 influential BMJ papers over the last 20 years [15]. Wieringa and Greenhalgh [11] found in a systematic review of “mindline(s)” conducted 10 years after the publication that a few studies empirically explored mindlines. They argued key components of mindlines—such as knowledge coconstruction and shared sense-making—need further research. Mindlines are formed in teaching hospitals as trainees learn how to think like clinicians [12].

The studies of Gabbay and le May [12,13] and Timmermans and Angell [14] occurred before smartphones and advanced Web-based systems exponentially increased accessibility of information. More recent studies assessed the impact of smartphones and information retrieval technologies on primary care clinicians [16,17] and internal medicine residents [18], arguing that these technologies increase the degree and variety of evidence accessed by clinicians. Others have assessed how virtual networks of clinicians inform the development of mindlines [19]. None of these studies observed how clinicians used scientific evidence in their daily work. This paper explores the ways that scientific evidence was used in the context of everyday practice and how clinicians and trainees draw on a multiplicity of knowledge in their decision-making processes in the age of smartphones and Web-based information systems. Its objective is to understand how evidence is integrated into mindline development and the everyday use of mindlines and evidence. It does so by exploring the ways clinicians teach trainees to use evidence in care, which sorts of evidence clinicians invoke from mindlines and which are read, and how evidence use changes as trainees develop their mindlines.

## Methods

### Design

This paper is based on an integrated knowledge translation project [20]. Hospital practitioners and administrators formed a project advisory committee, which advised during research design, data collection and analysis, and acted on project findings. The project focused on a problem identified by a health administrator—doctors did not follow CPGs. We used an ethnographic approach to study how evidence, including CPGs, was used in Internal Medicine at 2 teaching hospitals (identified as “Hospital A” and “Hospital B”). Multiple methods were employed, including ethnographic shadowing of care workers, in situ interviewing, and patient chart audits (reported elsewhere). This study received ethics approval from the Research Ethics Boards at the University of British Columbia (CREB#H15-0118) and Simon Fraser University (REB#2015s0388). Both Hospitals A and B granted organizational approval for this project. Informed consent was obtained from all participants directly shadowed or interviewed.

### Data Collection

Hospital care in Internal Medicine represents a complex, dynamic, and fast-paced environment. Attendings, residents, and medical students rotate between specialties and hospitals. Rotations range from 1 to 6 weeks, leading to high turnover as teams of workers are constantly being formed and reformed. Internal Medicine at both hospitals is called the Clinical Teaching Unit (CTU) as it is actively involved in training residents and medical students.

One of this paper’s authors, BL, shadowed care providers. Shadowing involved following and observing participants during their normal work and asking them, during lull periods, to interpret actions through informal interviews [21,22]. BL shadowed 6 CTU teams between October 2015 and January 2016 in 4 4-week blocks that alternated hospitals. Patients are predominately admitted to the CTU from the Emergency Department (ED). During the first 4-week block at each hospital, BL shadowed the CTU team—comprised an attending and 1 to 3 senior residents—involved in ED patient assessments and admissions as well as senior residents overseeing ED night admissions. During the next 4-week block at each hospital, BL shadowed 2 CTU teams—comprised an attending, a senior resident, 2 to 3 junior residents, and 2 to 4 medical students—involved in patient care on the wards during the day and ED patient admissions during their on-call night shifts. We refer to both residents and medical students as “trainees” unless a subgroup is identified.

All CTU members approached for this study agreed to participate. In total, 35 CTU team members consented to be shadowed or interviewed for the project. Many more CTU team members who were incidentally encountered during shadowing were recorded in fieldnotes. Shadowing occurred in 4-hour blocks to observe work processes while maintaining fieldnote quality. Shadowing was supplemented by a small number of observations of formal teaching during an annual CTU junior resident orientation. Table 1 outlines data collected at Hospitals A and B by data type, hours, location, and number of shifts. In total, BL collected 168 hours of data.

**Table 1:** Internal medicine team data collection in Hospitals A and B by type, hours, and number of shifts. Includes attending clinicians, residents, and medical students.

Type of data collected		Hospital A, hours (shifts)	Hospital B, hours (shifts)	Total, hours (shifts)
**Shadowing**				
	Emergency department	35 hours, 5 minutes (8)	58 hours, 38 minutes (13)	93 hours, 43 minutes (21)
	Internal medicine ward	28 hours (7)	38 hours, 20 minutes (13)	66 hours, 20 minutes (20)
Formal teaching observation		—^a^	4 hours, 15 minutes (3)	4 hours, 15 minutes (3)
Total		63 hours, 5 minutes (15)	101 hours, 13 minutes (29)	168 hours, 18 minutes (44)

^a^—: not applicable.

### Data Analysis

Fieldnotes were transcribed into more complete computer-typed notes at the earliest possible opportunity and imported into NVivo (qualitative data analysis software by QSR International). Analysis of data began during data collection. A key early insight was that CPGs were 1 of many evidence sources CTU members referenced during work. This became a focus of subsequent observations and fieldnotes. By reading fieldnotes, we created evidence categories: CPGs, peer-reviewed articles, UpToDate and Lexi-Interact, Google, Pocket Medicine, phone apps, and experience. References to evidence were also categorized by events (teaching or care) and role of person referencing evidence. See Multimedia Appendix 1 for a description of these categories.

We read fieldnotes tagging passages by evidence type, event, and role. Each passage could be tagged multiple times if different sources of evidence were referenced by CTU team members in discussion. Fieldnotes were reread and tags were checked. Using the NVivo query function, we created 2 matrices that counted tags of (1) evidence by event and (2) within care, evidence by role. These matrices were exported into R (the R foundation) [23] for further analysis. We also developed memos that used thick description and vignettes to describe categories.

Preliminary findings were outlined and presented to the project’s advisory committee at regular intervals for feedback. Emergent findings were also discussed with informants as a form of “member check” [24]. Results from quantitative category tags were triangulated memo descriptions and feedback to obtain a more holistic understanding of evidence use. All names used here are pseudonyms. Fieldnote excerpts refer to a fieldnote number followed by a line number (ie, 0116:23).

## Results

### Evidence Use in Teaching

**Figure 2:**
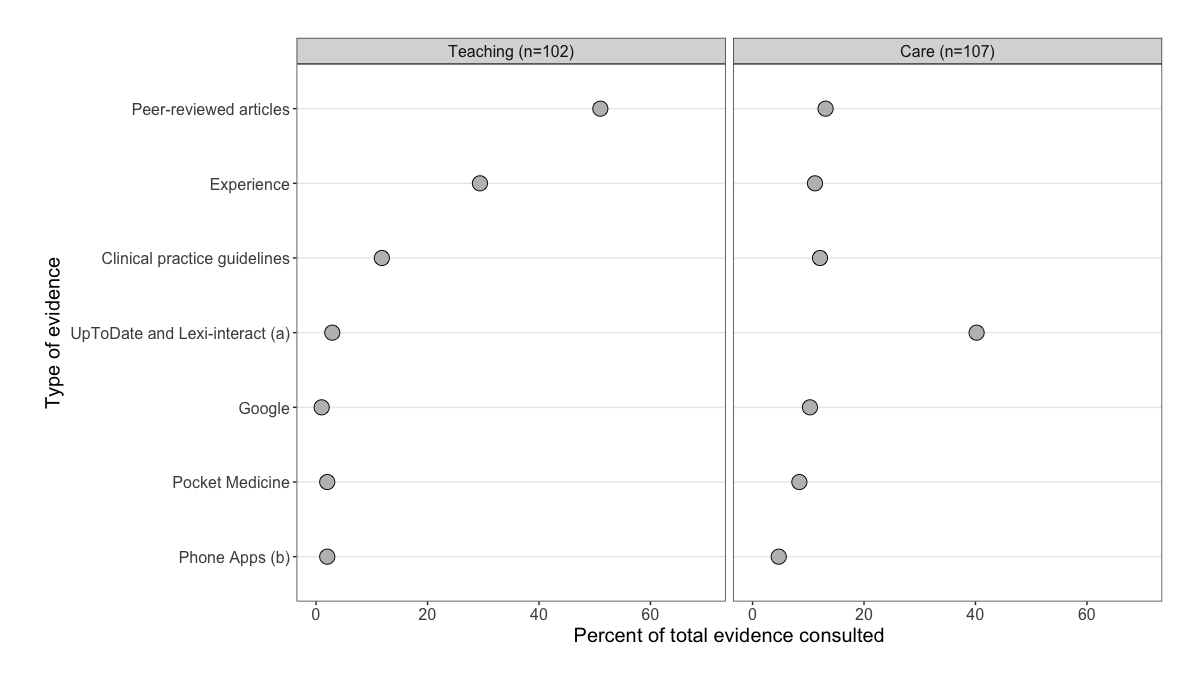
Observations of different types of evidence consulted in teaching and care; (a) includes UpToDate and Lexi-interact consulted through a phone app, (b) excludes UpToDate and Lexi-interact consulted through a phone app.

We found that scientific evidence was used differently in teaching and care. Figure 2 compares teaching and care events and presents counted evidence tags by category as a percent of total evidence tags in each event group. In teaching, the largest percent of counted evidence tags are peer-reviewed articles and experience. Our observations indicated that teaching sessions often began with the introduction of a patient case followed by a patient care plan informed by evidence:

Teal [senior resident] is teaching to a room of trainees. She begins by presenting a case where a pregnant woman has a fever, feels dizzy, falls over, and gets admitted to hospital. Over the next half hour, Teal[with input from the residents in the audience] writes out a strategy to initially manage, diagnose, and treat the patient for sepsis. One of the treatment strategies for sepsis is to give patients IV fluids. Teal tells the room that this patient got 2 liters of normal saline solution and asks the audience if that's a good amount to give or if they think the patient should get more or less. She continues, saying that “I want to challenge” the amount of fluids given to a septic patient. “We were all taught the Rivers Protocol.” Teal adds “The new approach is to give a certain amount of fluids and then reassess. We were all taught to keep on giving fluids but that is now changing because fluids given above a certain point may be associated with increased mortality.” Teal compares these recommendations to the UK sepsis guidelines for pregnant women and mentions that she couldn’t find any equivalent Canadian guidelines. She reminds the room of the mantra “healthy mommy is healthy baby” and that in these types of situations the focus should be on caring for the mother not the baby.

1028:30-174

Teal used this session to model evidence use that predominately draws from peer-reviewed articles and CPGs. Even though the trainees in the audience graduated from medical school within 3 years, Teal told them their knowledge is out of date as scientific evidence has evolved. In another sepsis teaching session, a Fellow (completing subspecialty training after his internal medicine residency) acting as a guest lecturer discussed the peer-reviewed articles that support the new sepsis approach and rhetorically asked if new evidence means the Rivers dogma is broken. A resident replied by asking the following question:

What protocol do we follow if we have a septic patient?

0721:187-466

The Fellow left this question unanswered.

In formal teaching, experience was often used to fill holes and cover grey areas in scientific evidence. These were cases where guidelines were not clear:

There are no clear guidelines, it’s a clinical decision.

Nicola, attending, 0107:148

A resident stated the following:

Treatments have been used for 100 years and they work so we use them

Ray, attending, 0719:151

Another resident stated the following about the treatment:

Is empiric, based on what people do not based on a clinical trial.

Adam, senior resident, 0219:65

### Invoking and Reading Evidence in Patient Care

In care, some types of evidence such as experience and peer-reviewed articles were predominately invoked from mindlines, whereas other sources such as UpToDate, Lexi-Interact, Pocket Medicine, and phone apps were read. CPGs were sometimes read and sometimes invoked. The following example illustrates how evidence was invoked in care:

Sara [attending] is reviewing a patient admission with Phil [medical student] in the ED. The rest of Sara's CTU team listens. The patient in question is an elderly man who is being admitted due to delirium and a fall. Phil begins to summarise the patient’s history and provisional diagnosis, listing delirium as the patient’s primary issue. He then begins to outline possible causes for the delirium using the DIMS mnemonic [drugs, infections, metabolic and structural, a mnemonic commonly used to treat delirious patients with no explicit grounding in research or guidelines]. Sara tells the team that “mine is DIMS UFO where U is urine, F is fecal, and O is more involved. We will go over it this afternoon during teaching but the U is based on a paper by [Sara gives the author's name] who is the Godmother of delirium.”

1215:89

In this example, Sara talked with trainees in a process to coconstruct an appropriate approach to caring for their patients. She made explicit her tacit thought processes and how what sorts of information were incorporated into her mindline.

Other evidence was read. Attendings would read from Lexi-Interact or UpToDate during conversations with trainees to double-check medication dosing, their inferences, and supplement gaps in their mindlines. Trainees explicitly searched and read evidence when developing care plans:

Fred [senior resident] stands outside of a patient’s [P2] room. Kate [medical student] comes out of P2’s room...Kate tells Fred that P2 grew e. coli in his nephrostomy tube [like a catheter but inserted directly into the kidney] and she thinks that they should prescribe antibiotics. Kate adds that she isn't sure which antibiotics to prescribe...Fred goes to UpToDate and types in the search terms “nephrostomy UTI” and then scans the first UpToDate document that comes up. He goes to the health authority’s formulary site and looks for antibiotics for catheter associated infection and skims the article and reads a section out loud to Kate saying 'do not treat positive cultures without symptoms.' Fred asks Kate if she has heard of CAUTI [catheter associated urinary tract infection] and searches for, then shows Kate, a page on UpToDate about the condition. Fred adds that he’s not sure if the same approach would apply to a nephrostomy infection. Fred then Googles nephrostomy and infection and finds an article by the Infectious Diseases Society of America about nephrostomy and infection. Kate smiles at Fred and tells him that she likes that organisation. They skim the article together but it has no recommendations about what they should do if they get a positive culture. Kate takes her IPad out and begins to search for information as well. She finds another entry in UpToDate that says that cultures in asymptomatic patients shouldn't be treated but that the tube should be changed as soon as possible. Fred looks at P2’s lab results. P2 has no white blood count and he is afebrile. Kate adds that she worries about P2 because he is immunocompromised...Fred decides that they won’t treat for the positive culture but that they might try and get P2’s tube changed.

0105:431

In this case, Fred and Kate sifted through various sources of evidence and personal knowledge together to make sense of a clinical problem and develop a care plan. Fred drew from his own knowledge of how to treat catheter associated urinary tract infections, but he was unsure if nephrostomy associated infections were similar enough for his knowledge to be applicable. Both Fred and Kate read to supplement what they know, using computers and iPads to access information on the Web. The information selected for reading was not peer-reviewed articles but was summaries of existing evidence with concrete recommendations. Multiple Web-based sources were skimmed for relevant information. Through discussions, Fred and Kate combined the knowledge extracted from summary documents, their nascent mindlines, patient laboratory results, and their knowledge of the patient to form a provisional care plan for P2.

CPGs were invoked or read. For example, CPGs were invoked when 2 patients were admitted to the CTU for carbon monoxide poisoning. Still in the ED, the patients were scheduled for 3 hyperbaric dives (where they would be placed in a chamber with high pressured oxygen to reduce the amount of carbon monoxide in their blood). Matt (attending) told BL that he believed the carbon monoxide level in both patients was low and he was not sure if the dives were necessary. Matt greeted the ED doctor overseeing the hyperbaric chamber and asked him if the hyperbaric dives were necessary. He explained that the patients’ carbon monoxide levels were already low and asked the following question:

How much does it help at this point?

Matt, attending

The ED doctor answered that current best practice said to do the 3 dives and added the following:

There is absolutely no evidence but it is standard of care so we’ll do it. We’ll do 3. They’ll get the benefits that they’ll get...the literature is unclear, no consistent evidence of benefit.

1023:372

Here, both Matt and an ED doctor made sense of an appropriate treatment plan for 2 patients. Matt questioned the agreed-upon approach, citing specific laboratory results for these 2 patients. The ED doctor invoked “best practice” to justify his own mindline for treating carbon monoxide poisoning. In this case, “best practice” was not viewed as evidence based. Rather, the ED doctor believed that following the best practice is a prudent approach in the absence of clear evidence. In other situations, trainees read CPGs to help inform care decisions. Local guidelines are one of several Web-based evidence sources used by Fred and Kate to formulate a care plan for P2.

### Developing Mindlines in Patient Care

**Figure 3:**
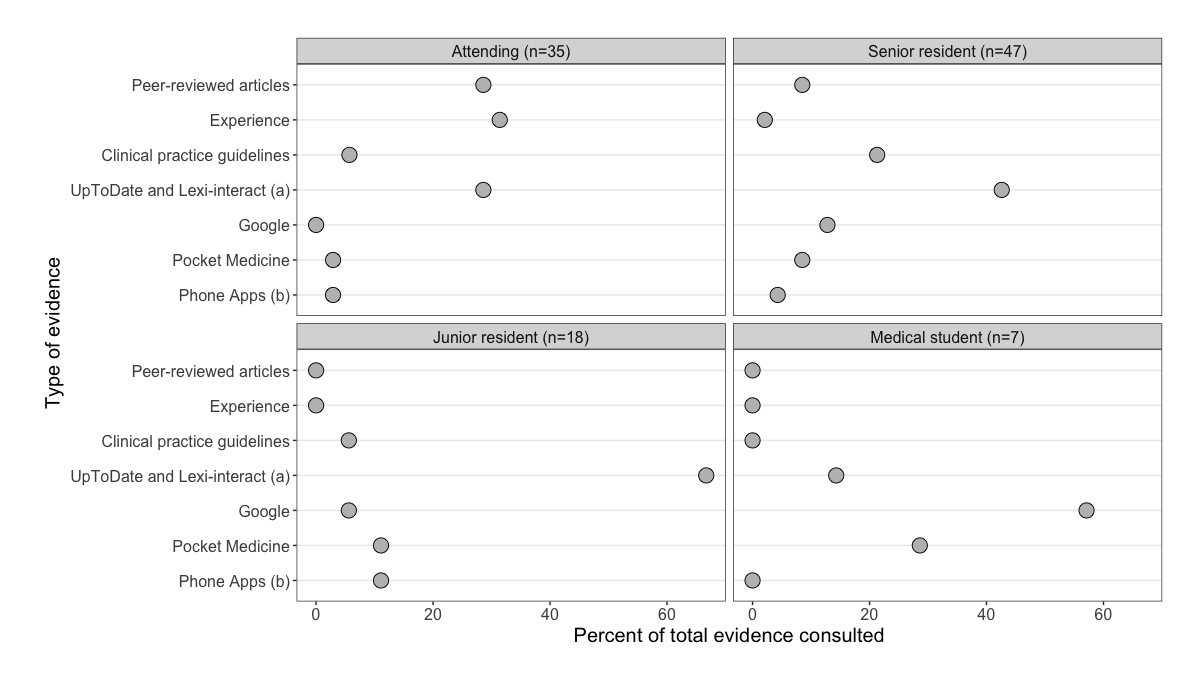
Observations of different types of evidence consulted in care by role; (a) includes UpToDate and Lexi-interact consulted through a phone app, (b) excludes UpToDate and Lexi-interact consulted through a phone app.

In care, evidence use varied by role. Figure 3 compares care roles and presents counted evidence tags by category as a percent of total evidence tags for each role. Figure 3 shows that individuals in all roles read UpToDate. Medical students and junior residents predominately read summary sources of evidence such as UpToDate, Lexi-interact, Pocket Medicine, and phone apps. Conversely, attendings predominately invoked their own experience or knowledge of peer-reviewed articles from their mindlines. Senior residents consulted the widest variety of evidence sources not only drawing on summary sources (particularly UpToDate) but also invoking peer-reviewed articles and experience. Senior CTU team members may be more capable of using their mindlines to filter scientific uncertainty than more junior trainees who are developing their mindlines. Consistent with the mindlines concept, it was widely recognized that clinical and scientific uncertainty often remain despite referring to a broad array of evidence and information and that clinicians may ultimately make choices on the basis of their interpretation of this uncertainty in the context of patient care needs.

We observed that trainees would often gather multiple kinds of information to develop a narrative of patient diagnosis and care that was presented to an attending. This formal presentation was used by attendings to review trainees’ reasoning processes and how knowledge of existing evidence was being incorporated into their developing mindlines. Attendings guided trainees on how mindlines were formed by quizzing them on how to think logically and identify and filter evidence and information when developing patient care plans.

## Discussion

### Principal Findings

This study explored the integration of evidence into mindline development and how mindlines and evidence are used. In this study, multiple forms of Web-based evidence were accessed—often through smartphones—and mixed with other forms of information. Evidence use varied between teaching and care and, within care, by role. Teaching modeled optimal evidence use and predominately drew on peer-reviewed journals and experience. In care, attendings’ evidence use most closely approximated the evidence use modeled in teaching. Some forms of evidence—such as experience, peer-reviewed journals, and some CPGs—were predominately invoked through mindlines. Other forms of evidence—such as UpToDate, Lexi-Interact, Pocket Medicine, and phone apps—were read to supplement mindlines. As CTU team members gained experience, they increasingly incorporated evidence into their mindlines. An alternative explanation to the observed variation of evidence use by role is a cohort effect whereby trainees are more comfortable accessing Web-based information systems and using smartphones. Although a cohort effect may explain some of the observed variance, it does not explain differences in how senior and junior residents (separated by a year) consult evidence.

In 1961, Becker et al [25] reported instances where experience was invoked to overrule scientifically verified knowledge—a phenomenon we did not observe. Although Gabbay and le May [12] state that they rarely observed care providers reading summary evidence to inform their daily practice, we observed this practice across all groups. These 3 studies may represent different stages in the institutionalization of the evidence-based medicine paradigm into clinical practice representing pre [25], early [12], and later (seen here) stages. Our observations may also be a result of the increasing codification of evidence in commercially produced apps and other tools and their ease of access on smartphones and computers. Gabbay and le May [12] noted that by the end of their observations, clinicians were consulting evidence more frequently. Many of the clinicians observed by Gabbay and le May [12] trained before 1990, when evidence-based medicine was introduced as part of medical school training, whereas our study occurred in teaching hospitals where evidence-based medicine is emphasized. Gabbay and le May [12] noted that UpToDate was referred to as a “bible” during their observations in a teaching hospital’s internal medicine specialty.

The sepsis example illustrates that the implications of evolving scientific knowledge on daily clinical practice are not always explicit [14]. CTU team members were also cognizant that existing evidence is occasionally of low quality, can make competing claims, and may not cover a clinical problem.

Our research suggests that evidence-based medicine has been incorporated into the work of the CTU teams studied here, just not in the ways envisioned by health administrators and policy makers who frequently equate low CPG uptake and a lack of standardization with not practicing evidence-based medicine. Instead of drawing on CPGs, CTU teams invoke and read a variety of evidence sources, blending them together to arrive at shared understandings of patient care [19,26,27]. As clinicians increasingly rely on their smartphones to access information, health administrators and policy makers will decreasingly be able to control the type and quality of information they access. The ways that managers assess and support clinician use evidence need to be expanded. It is important that these players understand current use patterns to support the application of quality evidence into clinical practice decisions.

### Limitations and Future Research

Many insights gained from shadowing internal medicine teams were based on a small number of teams at 2 hospitals, which limits generalizability. There is a chance that participants increased their use of evidence as they knew they were being shadowed (ie, Hawthorne effect). To decrease this impact, BL maintained a neutral attitude throughout her observations, emphasized that her primary interest was to understand how work was conducted, and shadowed an internal medicine team for at least 1 week.

Ethnographic research enables a rich understanding of the complex world around us. Writing fieldnotes necessarily distills from this complexity. Observing evidence use was a primary objective in these fieldnotes, but it is probable that not all instances where evidence was used were captured. By observing in depth, ethnographic work cannot capture the same breadth of data as other methodologies. The numbers for some observations—such as counts of evidence use tags by junior residents and medical students in care—are small. Specific numbers or detailed analyses of variance are not provided in the text as counts cannot be considered exact. Rather, they represent trends that were combined with memos and general observations and then checked with informants and experts to derive insight. Additional quantitative studies that track information use on phones and computers are essential to validate findings.

UpToDate, Lexi-Interact, and Pocket Medicine are all published by the same private company. Combined, they represent over half of the evidence we observed being consulted. An avenue of future research is to compare the recommendations provided by this company to other sources of evidence to understand if a bias is being created in how medicine is practiced. It is unclear if the work practices observed in these 2 teaching hospitals have diffused into community-based hospitals and general practices. Further research is needed to understand whether CPGs and other tools such as order sets may be more effective vehicles to incorporate evidence into practice in other settings.

### Conclusions

This paper has responded to calls for more empirical work exploring the complex ways that evidence and mindlines are incorporated into teaching and care [4,11]. It shows how mindlines are enacted [11-13] as clinicians blend multiple forms of evidence together to arrive at shared understandings and approaches to patient care and understand how this process varies as trainees develop their mindlines.

## Appendix

Multimedia Appendix 1 Fieldnote categorization.

## Abbreviations

CPG: clinical practice guideline
CTU: clinical teaching unit
ED: emergency department
