# Supplementary material for: Exploring How Evidence is Used in Care Through an Organizational Ethnography of Two Teaching Hospitals
Source: J Med Internet Res. 2019 Mar 28;21(3):e10769. doi: 10.2196/10769 (PMC6458541; doi:10.2196/10769)
Supplement: Multimedia Appendix 1 [file jmir_v21i3e10769_app1.pdf]

Fieldnote Categorisation

Evidence Categories

Peer reviewed Articles: Predominately clinical trials published in high impact journals.

UpToDate and Lexi-Interact: UpToDate and Lexi-Interact are provided by the same private company on a subscription basis and can be accessed through computers or smart phones. UpToDate is an evidence-based, physician-authored clinical decision support service. Its website is searchable with over 10,500 topic reviews that are constantly being updated. Entries range from 10-40 pages and include overviews of conditions and recommendations for treatment. Entries synthesize and explicitly reference a variety of evidence including CPGs, clinical trials, and meta-analyses. Lexi-Interact is a drug database that identifies possible interactions—and its risk rating—between drugs and recommends responses to interactions. The following field note excerpt illustrates how UpToDate and Lexi-Interact are used:

> *Matt [attending] is reviewing a patient admission with a resident. He asks the resident if he has checked the drugs for the patient related to their kidney transplant. The resident shakes his head and Matt tells him to use UpToDate and that he's "checking on my phone." The resident asks Matt "what are you looking for?" Matt tells the resident to punch all of the drugs that the patient is taking into Lexi-Interact and adds that you need to remember that with transplant patients some drugs are super dirty and you have to check for interactions. (1007:306)*

Here Matt—an attending physician—models for a resident how to use an existing online information system to inform prescription choices for a complex patient.

Pocket Medicine: Pocket Medicine[1] is a book designed to fit in the pocket of a physician's white coat. Through bulleted lists, tables, and algorithms it provides diagnosis and treatment suggestions for the most common disorders in internal medicine:

> *Nate [senior resident] is writing in a history sheet about a patient that he is assessing for admission. He keeps on looking at his phone and [initials blinded] asks him what he is looking at. He tells [initials blinded] that he has a book called Pocket Medicine and that he took pictures of the different pages and put the pictures into his phone. Nate shows [initials blinded] the photographed pages on his phone. [initials blinded] can make out his own penciled notes on the book's pages in the photograph. Nate tells [initials blinded] that he used to carry the book around with him but decided that it was easier to take pictures of the different pages. Right now he's looking at the book's heart failure section and reviewing the book's recommendations for how to treat heart failure patients. (1016:233)*

Phone Applications: In the examples outlined above, both Matt and Nate rely on their personal smart phones to search for scientific evidence to solve clinical problems encountered in their practice. While Matt relies on a specialised application, Nate has adapted a physical book to an electronic form for ease of transport. BL often asked CTU team members what they were doing on their phones but, out of respect for their privacy, BL tried not to peek into their personal

phone activity unless invited to do so. Thus, references to phone applications often went unobserved.

Experience: We classified experience as an additional type of evidence. We recognise that experience is often shaped by evidence. Several existing studies, including the mindlines work of Gabbay and le May, argued that clinicians predominately drew on experience not evidence in their work[2–4]. We wanted to compare our findings to these studies.

Event Categories

Teaching: Included CTU orientation, lectures, and care discussions not referring to specific patients.

Care: Discussions referring to specific patients.

Role Categories

Attending Physician

Senior Resident: In the second or third year of their residency.

Junior Resident: In the first year of their residency.

Medical Student: Completed two years of coursework and in their second or their year of medical school.

References

1. Sabatine MS, editor. Pocket Medicine. 6th ed. Wolters Kluwer; 2016.

2. Pope C. Resisting evidence: the study of evidence-based medicine as a contemporary social movement. Health (N Y) 2003;7(3):267–282.

3. Gabbay J, le May A. Evidence based guidelines or collectively constructed “mindlines?” Ethnographic study of knowledge management in primary care. Bmj 2004;329(7473):1013.

4. Becker HS, Geer B, Hughes EC, Strauss AL. Boys in white: Student culture in medical school. New Brunswick: Transaction Publishers; 2005.
